# Dissociating the Effects of Visual Similarity for Brand Names and Common Words

**DOI:** 10.5334/joc.397

**Published:** 2024-08-28

**Authors:** Francisco Rocabado, Melanie Labusch, Manuel Perea, Jon Andoni Duñabeitia

**Affiliations:** 1 Centro de Investigación Nebrija en Cognición (CINC), Universidad Antonio de Nebrija, Madrid, Spain; 2 Departamento de Metodología and ERI-Lectura, Universitat de València, Valencia, Spain

**Keywords:** word identification, lexical access, brand names, abstractionist accounts

## Abstract

Abstractionist models of visual word recognition can easily accommodate the absence of visual similarity effects in misspelled common words (e.g., *viotin* vs. *viocin*) during lexical decision tasks. However, these models fail to account for the sizable effects of visual similarity observed in misspelled brand names (e.g., *anazon* produces longer responses and more errors than *atazon*). Importantly, this dissociation has only been reported in separate experiments. Thus, a crucial experiment is necessary to simultaneously examine the role of visual similarity with misspelled common words and brand names. In the current experiment, participants performed a lexical decision task using both brand names and common words. Nonword foils were created by replacing visually similar letters (e.g., *anazon* [baseword: *amazon*], *anarilllo* [amarillo, yellow]) or visually dissimilar letters (e.g., *atazon, atarillo*). Results showed sizeable visual letter similarity effects for misspelled brand names in response times and percent error. Critically, these effects were absent for misspelled common words. The pervasiveness of visual similarity effects for misspelled brand names, even in the presence of common words, challenges purely abstractionist accounts of visual word recognition. Instead, these findings support instance-based and weakly abstractionist theories, suggesting that episodic traces in the mental lexicon may retain perceptual information, particularly when words are repeatedly presented in a similar format.

To efficiently recognize written words despite their variations in form, most models of reading and word recognition rely on the assumption that perceptual characteristics of a word’s letters (e.g., font, size, color, and letter CaSe) are rapidly abstracted via a normalization process (i.e., abstractionist theories; see Dehaene et al., 2005; Grainger et al., 2008 for biologically-plausible models; see Grainger, 2018; Grainger & Dufau, 2012, for reviews). Although surface elements may influence the initial moments of visual word recognition, as evidenced by their impact in early electrophysiological components in masked priming experiments (e.g., N/P150 component), their effect dissipates rapidly (see Chauncey et al., 2008, Vergara-Martínez et al., 2015, for early effects of size, font, and letter case).

Expanding on the findings supporting abstractionist theories, another key phenomenon favoring these theories is that in lexical decision tasks, responses to a misspelled word like *viotin* (visually similar to *violin*) and *viocin* (the visual overlap with *violin* [l/c] is smaller) are very similar in reaction times and error rates (Perea & Panadero, 2014; Perea et al. 2022) and elicit comparable electrophysiological activity (Gutiérrez-Sigut et al. 2022). If surface codes were major players during lexical access, *viotin* would be more similar to *violin* than *viocin*, yielding longer “no” decisions and more false alarms to *viotin*.

However, a specific category of words presents a significant challenge to the generality of abstractionist accounts of word recognition: brand names. Gontijo et al. (2002) found that plain brand names (i.e., without graphical design) are identified faster in a lexical decision experiment when presented in their usual letter-case format than in an uncommon letter-case format (e.g., *SAMSUNG* faster than *samsung*; *adidas* faster than *ADIDAS*; see also Perea et al., 2015). Similarly, when participants have to decide whether a given logotype was spelled correctly, responses are faster when presented intact (e.g., 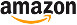) than when written in another typeface (e.g., 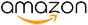) (Perea et al., 2021, see also Labusch et al., 2024a, for evidence with a semantic categorization task). Abstractionist theories of visual word recognition cannot easily capture these findings, as they would predict a null effect.

Of relevance to the present paper, an extremely challenging finding for purely abstractionist theories is that, unlike common words, visual letter similarity effects are sizeable for the misspellings of brand names and logotypes. When participants had to decide whether a logotype is real or not, Pathak et al. (2019) found longer response times and more errors for misspelled logotypes like *anazon* (visually similar to the actual logotype [*amazon*]) than *atazon* (visually dissimilar). Perea et al. (2022) replicated this effect with misspelled brand names both embedded in their logotypes and presented in plain text using the Times New Roman font—critically, the magnitude of the visual similarity effect was comparable across the two formats. This latter finding implies that elements of logotypes such as color, font, or design, are not crucial in driving the visual letter similarity effects for misspelled brand names. If this had been the case, one would have expected a larger visual letter similarity effect for logotypes than for brand names in plain text. In a second experiment, Perea et al. (2022) did not find a visual letter similarity effect for (misspelled) common words (e.g., *amarillo* (visually similar to the Spanish word *anarillo* [yellow] versus *atarillo* (visually dissimilar), suggesting different processing mechanisms for brand names than for common words. Naturally, the question arises as to which are the underlying properties that make brand names different from other common words.

The dissociation between the identification of misspelled common words and brand names could be explained in terms of instance-based theories of word recognition (e.g., Ans et al., 1998; Goldinger, 1998; Jacoby & Hayman, 1987; Jamieson et al., 2022; Kwantes & Mewhort, 1999; Reichle et al. 2022; Tenpenny, 1995). For brand names, design, font, and letter case remain consistent (i.e., as logotypes) across instances or episodes. For example, the brand name *IKEA* is habitually encountered in uppercase, with a characteristic bold typeface, and in a blue and yellow design (i.e., as in the logotype 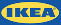). As a result, the memory traces created by brand names are mainly consist episodes in this format (e.g., the format of the *IKEA* logotype). After all, logotypes are designed to be easily memorable and rapidly accessible (Gontijo & Zhang, 2007). The result is that identifying brand names—or other common words that occur in particular contexts (e.g., the word *rosebud*; see Goldinger, 1998) would rely on episodic retrieval of the instances. On the other hand, common words are encountered in many visual formats (e.g., house, HOUSE, house, *house*, etc.) and contexts. Unlike brand names, one cannot quickly think of a specific font or letter case for a common word like *house*. Thus, proponents of episodic theories of lexical access have claimed that, while common words could be retrieved episodically, their representations would barely be affected by surface information of the individual exemplars (Goldinger, 1998; Tenpenny, 1995; see also Bowers, 2000, for a weakly abstractionist explanation of these effects).

A notable advantage of instance theories of word recognition is their integration with general frameworks in cognitive psychology, allowing for a comprehensive understanding of word processing across different domains (see Jamieson et al., 2022, for a recent proposal). However, before embracing these ideas in the context of visual word recognition, it is important to gather more conclusive evidence on the role of perceptual elements in the identification of brand names (see Bowers, 2000, for a critical view). Indeed, an interpretive issue in the visual letter similarity experiment with brand names reported by Perea et al. (2022) is that these items were presented alongside logotypes (e.g., *amazon* [*anazon, atazon*] could be presented in plain text or embedded in its logotype). This setup, in which logotypes *and* brand names appeared during the experiment, could have biased participants’ attention towards surface elements, thus making the brand names more memorable via episodic traces. More decisive evidence would require testing, within the same experiment, whether the effects of visual letter similarity on misspelled brand names occur in a stimulus list that contains common words, for which one would not expect any effect in either instance or abstractionist theories. To that end, in the present experiment, the two types of stimuli were presented within the same block; therefore, participants could not anticipate whether the following trial was a brand name or a common word. Note that Perea et al. (2022) presented these stimuli in separate experiments—(misspelled) brand names in Experiment 1 and (misspelled) common words in Experiment 2.

The predictions for the experiment are straightforward. If the identification of brand names, but not common words, is sensitive to perceptual codes—as measured by visual letter similarity effects in a lexical decision task, we would expect an interaction between type of misspelled stimulus (brand name, common word) and letter similarity: (1) a sizeable visual similarity effect for misspelled brand names (e.g., *anazon* producing more errors and longer response times than *atazon*) and (2) a negligible visual similarity effect for misspelled common words (e.g., *anarillo* and *atarillo* producing comparable response times and error rates). This outcome would support instance-based over abstractionist theories, thereby generalizing the findings reported by Perea et al. (2022) in a single experiment. Conversely, the absence of this interaction would suggest that the previous demonstrations of the visual similarity effect for brand names (e.g., Pathak et al., 2019; Perea et al., 2022) were not due to their mental representations but rather to their pairing with items embedding graphical information.

## Method

### Participants

Following the OSF preregistration, we recruited 68 native Spanish speakers from Spain with normal/corrected vision and no reading difficulties (M = 35.25 years, SD = 11.17; 42 males). This sample size ensured 4,080 observations per condition—in line with Brysbaert and Stevens’ (2018) guidelines. Recruitment was carried out through Prolific Academic (http://prolific.ac). This research received approval from the Research Ethics Committee at the University of València following the principles outlined in the Declaration of Helsinki. All participants provided informed consent and received a small monetary compensation.

### Materials

We employed the same base words as Perea et al. (2022). This set included ten popular brand names in Spain, presented in their usual letter case (amazon, Colgate, Google, intel, LACOSTE, Levi’s, DISNEY, MERCADONA, NESCAFÉ, and SAMSUNG) and ten common words (amarillo, delgado, PERSONA, negocio, sentido, FRACASO, nivel, MERCADO, DIFÍCIL, and COMERCIAL). We maintained the same pattern of letter case for the items in the two sets (e.g., *amazon* and *amarillo* would be presented in lowercase; *NESCAFÉ* and *DIFÍCIL* would be in uppercase). The replaced letters for the visually similar and visually dissimilar pseudowords were the same for the two sets (e.g., the brand name *amazon* would be used to create *anazon* and *atazon*, whereas the word *amarillo* [yellow] would be used to create *anarillo* and *atarillo*). In the Simpson et al. (2012) matrix, the mean visual similarity for the visually similar and visually dissimilar letters was 4.7 and 1.3 (out of 7), respectively. With one exception, the visually similar and dissimilar pseudowords were the same as in the Perea et al. (2022) experiments. Following the preregistration protocol, we substituted the misspelled brand name Colmate (brand: Colgate) as it could be interpreted as a combination of “Col “and “mate”—both of which are legitimate words in Spanish. We employed Colzate instead. For consistency, we did the same for its corresponding parallel item for common words (i.e., the pseudoword Delmado was replaced with Delzado). All stimuli were presented in Times New Roman font, maintaining their respective or assigned letter-case configuration.

### Procedure

**Figure 1:**
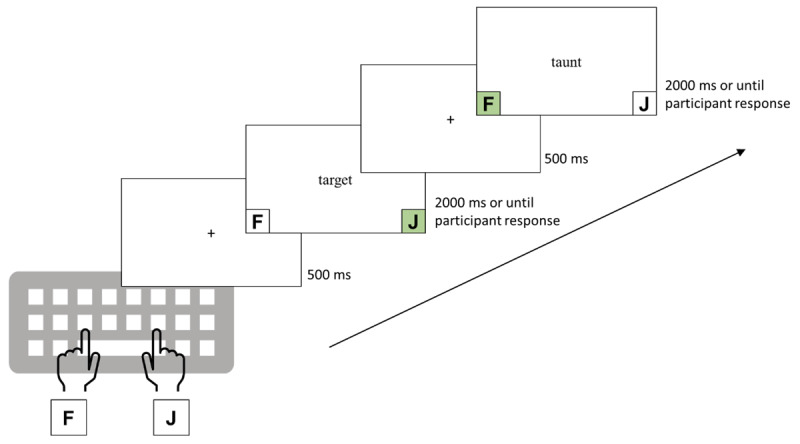
Scheme for a typical trial sequence in the experiment.

The experimental setup, design, and data collection were conducted using Gorilla Experiment Builder (Anwyl-Irvine et al., 2020, https://www.gorilla.sc). Participants were instructed to complete the experiment on a computer in a quiet room without distractions, following standard practices for online experimentation. Initially, participants provided their demographic information (e.g., age, gender identity, vision-related medical conditions). Afterward, participants were given the following instructions: press the J key if the presented stimulus corresponded to an actual word and press the F key if the presented stimulus was not an actual word. We presented a list of word stimuli, including brand names and common words, before the task to partially mitigate the saliency and potential recency effects associated with viewing the brand names—for a schematic trial representation, see Figure 1.

A 20-trial practice phase preceded the experimental phase to familiarize participants with the task. The setup was modeled after the experiments conducted by Perea et al. (2022), except that brand names and common words were presented in a single experiment instead of two separate experiments. We employed three blocks of 160 experimental trials each, totaling 480 trials (240 words [120 brand names, 120 common words]; 120 visually similar pseudowords [60 derived from brand names, 60 derived from common words]; 120 visually dissimilar pseudowords [60 derived from brand names, 60 derived from common words]). All items within a block were presented in a randomized order; self-paced rest breaks were provided after every 80 trials. The experiment lasted approximately 15 minutes.

### Data analysis

To analyze the response times and accuracy data for the critical items (i.e., the pseudowords), we created Bayesian linear mixed-effects models using brms (Bürkner, 2017) in the R environment (R Core Team, 2022). The response times were fitted with the exgaussian distribution (Ratcliff, 1993), and accuracy was fitted with the Bernoulli distribution (1 = correct; 0 = incorrect). The two fixed factors in the design were the type of base stimulus in the misspelled words (common word, brand name; coded –0.5 and 0.5) and visual letter similarity (similar, dissimilar; coded 0.5 and –0.5). The random-factor structure for both subjects and items in the models was set to the maximum allowed in the design:

RT(accuracy)=stimulus_type*visual_similarity+(stimulus_type*visual_similarity|subject)+(visual_similarity|item)

**Figure 2:**
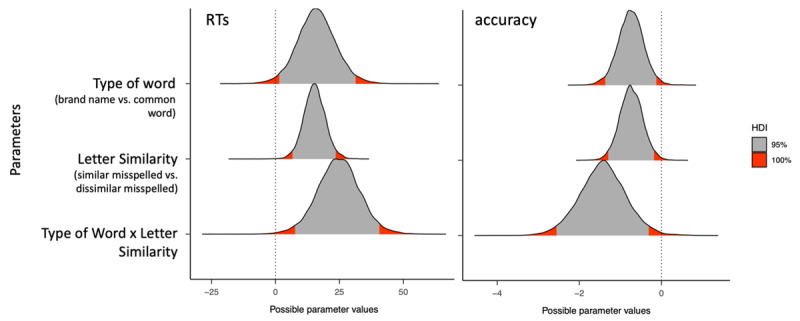
Posterior distributions of the response time and accuracy data. Deviations from the 95% Credible Intervals are marked in red. The acronym HDI refers to the Highest Density Intervals of the posterior distributions.

We ran 5000 iterations of each model—1000 for warmup—using four chains. All four chains converged adequately (R-hat = 1.00 for all parameters). In the output, Bayesian linear mixed-effects models indicate the parameter estimation corresponding to the mean of the posterior distribution. The models also provide the estimation error of each estimate, and the 95% Credible Interval (95%CrI) of its posterior distribution (see Figure 2 for the posterior distributions of the response time and accuracy data). When the 95% CrI of the parameter estimate did not overlap with zero, this was interpreted as evidence of an effect (i.e., a credible difference). We employed the package emmeans (Lenth et al., 2018) to examine simple test effects in case of evidence of an interaction between the two factors.

## Results

For the statistical analyses of the RTs, we excluded error responses (4.95% for pseudowords, 4.82% for words) and response times faster than 250 ms (less than 0.001%). Table 1 presents the average response times and error rates (in percentage) for each of the four conditions.

**Table 1:** Mean response times (in ms) and error rates (in percentages) for words, visually similar pseudowords, and visually dissimilar pseudowords in the experiment.


	BRAND NAMES		COMMON WORDS	
	
	RESPONSE TIME	ERROR RATE	RESPONSE TIME	ERROR RATE

Words	662	5.5	652	4.1

Visually Similar Pseudowords	713	11.6	668	3.2

Visually Dissimilar Pseudowords	661	2.5	650	2.5

*Visual Similarity Effect*	52	9.1	18	0.7


### Analysis of the Response Times

The latency data revealed faster responses to the visually dissimilar than to the visually similar misspellings (*b* = 15.25, SE = 4.25, 95%CrI [6.90, 23.78]) and faster responses to misspelled common words than to misspelled brand names (*b* = 16.06, SE = 7.52, 95%CrI [1.41, 31.30]). More importantly, we found evidence of an interaction between the two factors (*b* = 24.66, SE = 8.34, 95%CrI [7.94, 41.05]), reflecting a sizeable effect of visual similarity for misspelled brand names (i.e., longer RTs for visually similar misspellings: *b* = –27.64, 95%CrI [–39.5, –15.79]) but not for misspelled common words (*b* = –2.88, 95%CrI [–14.6, 8.86]) (see the Supplementary materials for complementary analyses using delta plots).

### Analysis of the Accuracy Data

We found a higher error rate for visually similar than for visually dissimilar misspellings (*b* = –0.74, SE = 0.28, 95%CrI [–1.29, –0.17]) and for misspelled brand names than misspelled common words (*b* = –0.75, SE = 0.31, 95%CrI [–1.38, –0.13]). Importantly, we found evidence of an interaction between both factors (*b* = –1.43, SE = 0.57, 95%CrI [–2.55 –0.30]), showing a visual letter similarity effect for misspelled brand names (i.e., lower accuracy for visually similar misspellings; *b* = 1.45, 95%CrI [0.644, 2.187]), but not for misspelled common words (*b* = 0.02, 95%CrI [–0.775, 0.852]).

## Discussion

Recent research has provided compelling evidence of a sizeable effect of visual letter similarity for misspelled brand names (e.g., *anazon* producing slower and more error-prone responses than *atazon* in lexical decision), challenging the dominant class of abstractionist models of visual word recognition. The main aim of the present experiment was to examine in the same experimental setup the effects of visual letter similarity for misspelled common words and brand names. Results showed a large effect of visual similarity for misspelled brand names but not for misspelled words. Thus, the effects of visual letter similarity with misspelled brand names in previous experiments (Pathak et al., 2019; Perea et al., 2022) were genuine and not due to list composition or task strategies.

While abstractionist models of visual word recognition can readily capture the null effects of visual letter similarity for misspelled common words, these models cannot explain the sizeable effects for brand names. These models would assume an early normalization process where perceptual codes are abstracted out for both types of stimuli (Dehaene et al., 2005; Grainger et al., 2008). Instead, the dissociation between the effects for brand names versus common words favors weakly abstractionist and instance theories of word recognition (Goldinger, 1998; Reichle et al., 2022, for instance-based accounts of lexical access). In instance-based models, the traces corresponding to lexical entries result from the myriads of encounters with each word. As common words have a long history of previous presentations in countless visual forms and contexts, there would be little episodic information in their stored representations, hence, they may appear as if they were “functionally abstract” (Goldinger, 1998, p. 268). This account can explain the comparable processing of misspelled words like *viotin* (visually similar to violin) and *viocin* in ERP (Gutierrez-Sigut et al., 2022) and behavioral experiments (e.g., Perea et al., 2022)—including the current one. The scenario is different for brand names, which are usually presented in a uniform format (e.g., as logotypes) and for which their mental representations may contain surface information (see Labusch et al., 2024a, 2024b, Rocabado et al., 2023, for discussions). As a result, in a task that requires to decide whether the stimulus is a word/brand name, it would be more difficult to say “no” to the visually similar pseudoword *anazon* than to the visually dissimilar pseudoword *atazon*.

Similarly, weakly abstractionist accounts can also explain the present findings. These models assume that visual word recognition is driven by a dynamic interplay between abstract and instance-based processes (see Bowers, 2000; Marsolek, 2004; Tenpenny, 1995). While most common words would mainly be processed in an abstract manner, perceptual information would play a role in those words that are typically presented in the same visual format (e.g., in brand names). In this way, weakly-abstractionist accounts align with the idea that specific visual features such as letter similarities may be considered during initial processing, alongside higher-level abstract representations (see also Marsolek, 2004; Schacter et al., 2004, for a parallel view within the framework of multiple-systems accounts).

Importantly, the idea of visual word recognition via retrieval through episodic memory traces has previously been captured by computational models of written word processing, such as the multiple-trace memory model (Ans et al., 1998) or the LEX model (Kwantes & Mewhort, 1999). Future versions of these models require further attention toward the specific mechanisms behind perceptually more uniform words like brand names—and likely other types of stimuli with some special characteristics like proper nouns, city names, or acronyms (e.g., see Henderson & Chard, 1976; Labusch et al., 2022; Perea et al., 2024; Peressotti et al., 2003; Sulpizio & Job, 2018; Wimmer et al., 2016), including the role of uniformity in the formats and whether the items are presented only in specific contexts (Goldinger, 1998). The present paper provides an initial step in this direction.

Altogether, the present lexical decision experiment has shown sizeable effects of visual letter similarity for misspelled brand names (i.e., *anazon* produced longer responses and more errors than *atazon*) and negligible effects for the parallel manipulation with misspelled common words. While this pattern is challenging for abstractionist theories of word recognition, instance theories and weakly abstractionist theories, which have been usually overlooked (but see Ans et al, 1998; Kwantes & Mewhort, 1999, for exceptions), provide an elegant explanation for this dissociation. Furthermore, instance theories fit well with many other phenomena in cognitive psychology (see Jamieson et al., 2022). Future research should examine the limits of abstractionist vs. episodic accounts for the recognition of brand names and common words in scenarios other than laboratory word identification tasks. For instance, as suggested by a reviewer, a potential manipulation to directly test these accounts could be to run a word learning experiment that manipulates the format of the words (constant vs. variable) and the context in which these words appear (isolated vs. in context).

## Data Accessibility Statement

This experiment was pre-registered at the following OSF link: https://osf.io/nbywz/?view_only=d2827286e713444db268691c2804c916

The OSF link with the script, data, and output is: https://osf.io/8gt2m/?view_only=d50c015004aa4388a8be72082318a7f0

## Additional File

The additional file for this article can be found as follows:

10.5334/joc.397.s1 Supplementary Materials. Complementary analyses using delta plots.
