# Supplementary material for: Dissociating the Effects of Visual Similarity for Brand Names and Common Words
Source: J Cogn. 2024 Aug 28;7(1):67. doi: 10.5334/joc.397 (PMC11363898; doi:10.5334/joc.397)
Supplement: Supplementary Materials. — Complementary analyses using delta plots. [file joc-7-1-397-s1.pdf]

Supplementary Materials

As in the Perea et al. (2022) experiments, we conducted an exploratory analysis via delta plots to further explore the dissociation between the visual similarity effect for misspelled brand names and common words (see Ridderinkhof, 2002). Thus, for misspelled brand names and common words, we computed the by-subjects average of the .1, .3,…, .9 quantiles of the visual similarity effect (i.e., the difference in RTs between visually similar and visually dissimilar items). On the one hand, an approximate flat line around zero ms would reflect no differences in visual letter similarity, and an approximate flat line around 40 ms would reflect a comparable effect of visual letter similarity across quantiles. This outcome would represent a shift in the RT distributions, reflecting a difference in encoding/response time (Ratcliff, Gomez, & McKoon, 2004). On the other hand, an increased effect of letter similarity across quantiles (i.e., more skewed RT distribution for the slower condition) would reflect differences in the quality of evidence (Ratcliff, Gomez, & McKoon, 2004).

For misspelled brand names, we found a sizeable effect of visual letter similarity that increases across quantiles (see the left panel of Figure 3). In contrast, for misspelled common words, the effect of letter similarity was quite small—it only increased in the higher quantiles (see the right panel of Figure 3). All in all, this pattern resembles that reported by Experiment 1 (brand names) and Experiment 2 (common words) by Perea et al. (2022). For the interested readers, we have included in the OSF link the parallel delta plots as a function of each block of the experiment (block 1 [trial 1-160], block 2 [trial 161-320], block 3 [trial 321-480]) which, as in the Perea et al. (2022) experiments, show a stable pattern across blocks.

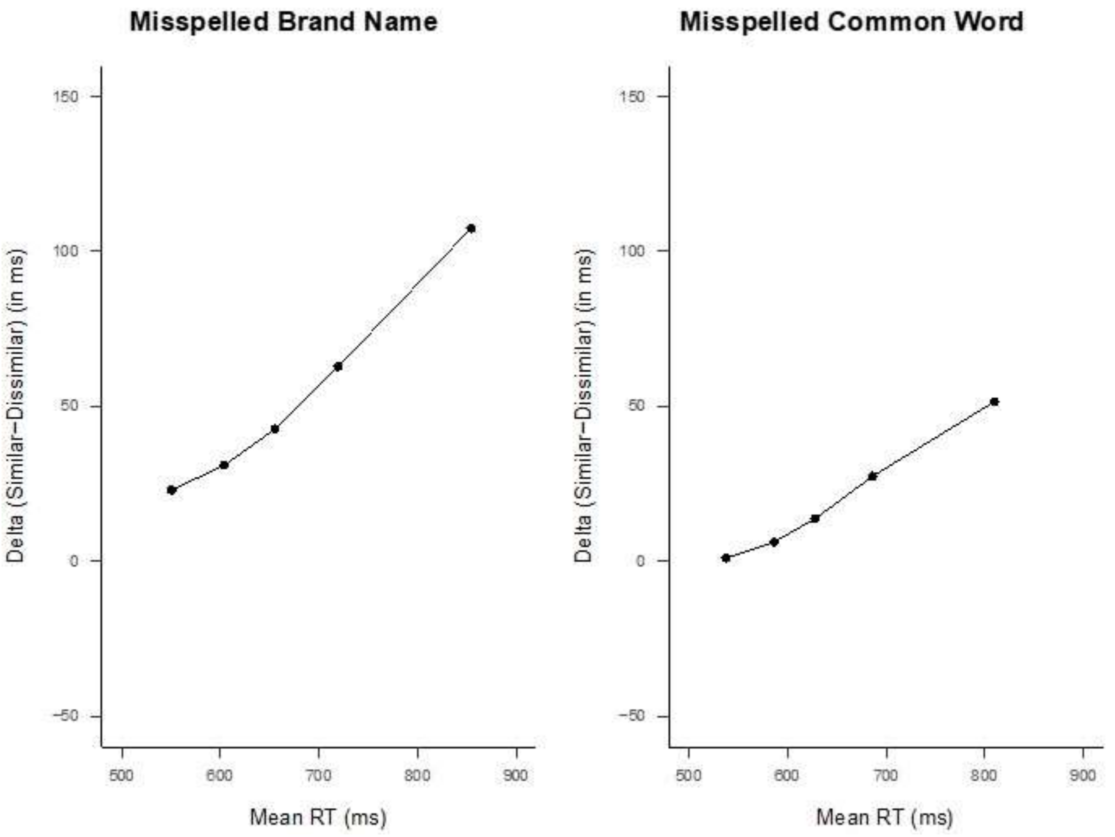

Figure 3. Delta plots of misspelled brand names (left panel) and misspelled common words (right panel) calculated by the difference between similar and dissimilar pseudowords across the .1, .3, .5, .7, and .9 quantiles.

References

Perea, M., Baciero, A., Labusch, M., Fernández-López, M., & Marcet, A. (2022). Are brand names special words? Letter visual similarity affects the identification of brand names, but not common words. *British Journal of Psychology, 113*, 835–852. https://doi.org/10.1111/bjop.12557

Ridderinkhof, K. R. (2002). Activation and suppression in conflict tasks: Empirical clarification through distributional analyses. In W. Prinz & B. Hommel (Eds.), *Common*

mechanisms in Perception and Action: Attention and performance XIX (pp. 494–519). Oxford University Press.

Ratcliff, R., Gomez, P., & McKoon, G. (2004). A diffusion model account of the lexical decision task. *Psychological Review, 111*, 159–182. https://doi.org/10.1037/0033-295X.111.1.159
